# CD3^+^/CD56^+^ NKT-like Cells Show Imbalanced Control Immediately after Exercise in Delayed-Onset Muscle Soreness

**DOI:** 10.3390/ijms231911117

**Published:** 2022-09-21

**Authors:** Balázs Sonkodi, Éva Pállinger, Tamás Radovits, Emese Csulak, Kinga Shenker-Horváth, Bence Kopper, Edit I. Buzás, Nóra Sydó, Béla Merkely

**Affiliations:** 1 Department of Health Sciences and Sport Medicine, Hungarian University of Sports Science, 1123 Budapest, Hungary; 2 Department of Genetics, Cell- and Immunobiology, Semmelweis University, 1085 Budapest, Hungary; 3 Heart and Vascular Center, Semmelweis University, 1122 Budapest, Hungary; 4 Faculty of Kinesiology, Hungarian University of Sports Science, 1123 Budapest, Hungary; 5 HCEMM-SU Extracellular Vesicle Research Group, 1089 Budapest, Hungary; 6 ELKH-SE Translational Extracellular Vesicle Research Group, 1089 Budapest, Hungary; 7 Department of Sports Medicine, Semmelweis University, 1122 Budapest, Hungary

**Keywords:** delayed-onset muscle soreness, NKT-like cells, Piezo2 ion channel, HSP-70, interleukin-6, NF-κB, NMDA

## Abstract

The purpose of the study was to carry out an immunophenotypical characterization with a special focus on natural killer cells of junior swimmers from the Hungarian National Swim Team before and after an intensive acute exercise. Nineteen swimmers, ten females and nine males, completed the exercise protocol. Sixteen swimmers experienced delayed-onset muscle soreness. Most of our findings substantiated earlier results, such as the increase in the percentage of the CD3^−^/CD56^+^ natural killer cells and the CD3^−^/CD56^dim+^ NK cells, and the decrease in the percentage of CD3^+^ T cells among lymphocytes after the exercise protocol. The drop of natural killer cell activity back to the pre-exercise level was in line with earlier findings. Interestingly, the percentage of CD3^+^/CD56^+^ NKT-like cells did not change significantly in those three swimmers who did not report delayed-onset muscle soreness. On the contrary, the percentage of CD3^+^/CD56^+^ NKT-like cells among lymphocytes increased in fourteen and decreased in two swimmers reporting delayed-onset muscle soreness. This study for the first time demonstrated a link between the delayed-onset muscle soreness and the imbalanced control of CD3^+^/CD56^+^ NKT-like cells among lymphocytes. However, validation of this association in a larger sample size study will be necessary.

## 1. Introduction

Delayed-onset muscle soreness (DOMS) is characterized by a delayed onset of soreness, muscle stiffness, swelling, loss of force-generating capacity, reduced joint range of motion, and decreased proprioceptive function [1]. Unaccustomed or strenuous eccentric contractions are observed to induce DOMS in association with the aforementioned neuromuscular changes [2,3,4]. The soreness of DOMS evolves after 8 h, peaks 1 to 2 days later [3], and diminishes within 7 days after exercise [5]. A number of theories have attempted to elucidate the pathophysiology of DOMS, such as with lactic acid, muscle spasm, connective tissue damage, muscle damage, and enzyme efflux theory [6]. However, scientists are consensual of favoring the notion that no single theory could disentangle this mysterious pathophysiology [6]. To date, efficient treatment options for DOMS are limited. However, interventions that are capable of increasing blood flow to the muscle, such as low-intensity concentric exercise, massage, or neurodynamic mobilization could alleviate the symptoms of DOMS, although this effect is often temporary [7,8,9].

Emerging studies are highlighting the role of reactive oxygen species production and oxidative stress in muscle damage, regeneration, and DOMS [10,11,12,13,14]. Earlier, the source of reactive oxygen species production and oxidative stress was mainly revealed in muscles [15]. However, a new study showed that intense acute swimming-derived DOMS induced oxidative stress in the spinal cord with resultant redox imbalance that contributes to nuclear factor-kappa B (NF-κB) activation and DOMS hyperalgesia [16].

A recent hypothesis postulated that the critical underlying damage in DOMS is neural within the muscle spindle [9]. This acute non-contact compression axonopathy theory puts forward that unaccustomed or strenuous eccentric contractions under an acute stress response could microdamage the primary afferent proprioceptive terminals in the muscle spindle [9]. This type of novel proprioceptive microdamage is learnt from chemotherapy and not associated with Wallerian degeneration experienced in peripheral nerve injury [17]. Even more recently, Piezo2 channelopathy was proposed as the most specific locus of the microdamage at the proprioceptive terminal [18]. This ion channel microinjury was hypothesized based on a strange medical condition called post-orgasmic illness syndrome (POIS), which has an unknown pathomechanism where the underlying damaging mechanism was suspected to be analogous with DOMS but in a chronic way [18]. One case study excluded that POIS was a hypersensitivity reaction and found a diminished number of CD56^dim^CD16^+^ natural killer (NK) cells [19]. Furthermore, some articles suggested an analogous underlying non-contact microinjury mechanism of the muscle spindle-derived proprioceptive terminals in DOMS and amyotrophic lateral sclerosis (ALS), as well [20,21]. However, in the case of ALS, regeneration from the proposed microinjury is non-resolving due to genetic predisposition and environmental risk factors [20,21]. Two genes have been known as major contributors to ALS pathophysiology, namely chromosome 9 open reading frame 72 and CuZn-superoxide dismutase genes, but many other genes are also associated with ALS [22]. Environmental risk factors comprise lifestyle and occupational risk factors, but various metals, pesticides, and viruses also show relationships with ALS [22]. Notably, ALS is associated with increased levels of natural killer T-like cells (NKT-like cells), diminished immune regulation by CD56^bright^ NK cells, and the importance of the NF-κB pathway activation [23,24,25]. Furthermore, it is important to note that the aforementioned present-day research indeed demonstrated that DOMS induces spinal neuroinflammation [16].

Knowledge about the neuroimmune consequences of a peripheral nerve injury with Wallerian degeneration is emerging, but the neuroimmune synapses of the proposed novel proprioceptive microinjury remains rather unexplored. Therefore, our current study aimed for an immunophenotypical characterization with a special focus on the NK cells of youth swimmers from the Hungarian National Swim Team prior to and post-exercise. The intense acute exercise protocol was specifically designed for swimmers and meant to induce DOMS. Regeneration of exercise-induced damages is highly dependent on inflammatory and immune responses. Various innate and adaptive immune cells coordinate this complex regeneration repair mechanism. NKT-like cells, for example, present both innate and adaptive immune cell characteristics. Mapping the immune responses of these cells to various exercise modalities and damages could be scientifically advantageous, especially in the time of the COVID-19 pandemic.

## 2. Results

### 2.1. Study Population

A detailed sports cardiology screening was carried out on 19 youth swimmers (Table 1). All the athletes were members of the National Swim Team Hungary and qualified for the Youth European Championships 2022. They trained about 20 h/week prior to the exercise test. There were no previous or present injuries nor any medical conditions that could link to musculoskeletal impairment. After the isometric weight holding task, 16 athletes reported DOMS.

### 2.2. CD3^−^/CD56^+^ NK Cells among Lymphocytes

**Figure 1:**
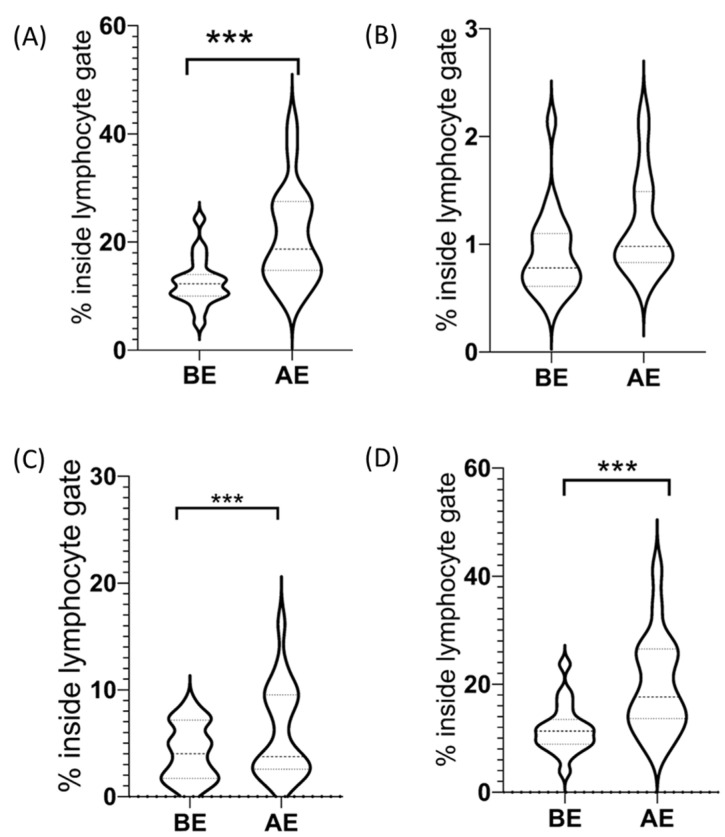
Frequencies of NK cell subsets. Violin plots show frequencies of NK cell subsets inside the lymphocyte gate before and after exercise: (**A**) CD3^−^/CD56^+^ NK cells, (**B**) CD3^−^/CD56^bright+^ NK cells, (**C**) CD3^+^/CD56^+^ NKT-like cells, (**D**) CD3^−^/CD56^dim+^ NK cells. *** *p*  <  0.001 (Wilcoxon test).

The percentage of CD3^−^/CD56^+^ NK cells among lymphocytes increased due to our exercise protocol in all our participants (before exercise (BE): 12.6 ± 1.01, after exercise (AE): 20.96 ± 2.04) (Figure 1). The increase was significant (65.8%, *p* < 0.05, ES = 1.08).

#### 2.2.1. CD3^−^/CD56^dim+^ NK Cells among Lymphocytes

The percentage of CD3^−^/CD56^dim+^ NK cells among lymphocytes increased due to our exercise protocol in all our participants (BE: 11.69 ± 1.04, AE: 19.79 ± 2.05) (Figure 1). This increase was significant (70.6%, *p* < 0.05, ES = 1.04).

#### 2.2.2. CD3^−^/CD56^bright+^ NK Cells among Lymphocytes

Considering all the participants in the study sample, the percentage of CD3^−^/CD56^bright+^ NK cells among lymphocytes did not change significantly (27.4%, *p* = 0.1) after the exercise protocol (BE: 0.91 ± 0.09, AE: 1.16 ± 0.10) (Figure 1). Furthermore, there was no correlation (*p* < 0.05) between subjects without DOMS and subjects with CD3^−^/CD56^bright+^ decline as a percentage of lymphocytes.

### 2.3. CD3^+^ T Cells among Lymphocytes

The percentage of CD3^+^ T cells among lymphocytes decreased due to our exercise protocol in all our participants (BE: 65.5 ± 6.6, AE: 58.1 ± 8.8). Our results indicate that the 12.7% decrease in the values after the exercise protocol was significant (*p* < 0.05, ES = 1.27).

#### CD3^+^/CD56^+^ NKT-like Cells among Lymphocytes

Considering all the participants in the study sample, the percentage of CD3^+^/CD56^+^ NKT-like cells among lymphocytes increased due to our exercise protocol (BE: 4.13 ± 0.59, AE: 6.14 ± 0.98) (Figure 1). Our results indicate that the 48.7% increase in the values after the exercise protocol was significant (*p* < 0.05, ES = 0.98, see Table 1). However, we must highlight that although CD3^+^/CD56^+^ NKT-like cells increased significantly as a percentage of lymphocytes due to our exercise protocol, the exceptions were those subjects who did not report DOMS. In these cases, the percentage of CD3^+^/CD56^+^ NKT-like cell frequency was steady among lymphocytes even after the exercise protocol. Furthermore, two of our subjects showed a decreasing CD^3+^/CD^56+^ NKT-like cell frequency due to our exercise protocol. Interestingly, if we evaluate the average of the differences between the before–after datasets of the individuals who reported DOMS after the protocol with the zero value using a one-sample t-test, we also get a significant difference (*p* < 0.05, ES = 1.16), indicating that the protocol in the subjects who reported DOMS did result in a change in the percentage of CD3^+^/CD56^+^ NKT-like cells. However, if we evaluate the average of the differences between the before–after datasets of the individuals who did not report DOMS after the protocol with the zero value, we do not find a significant difference (*p* = 0.75, ES = 0.2).

### 2.4. NK Cell Activity

**Figure 2:**
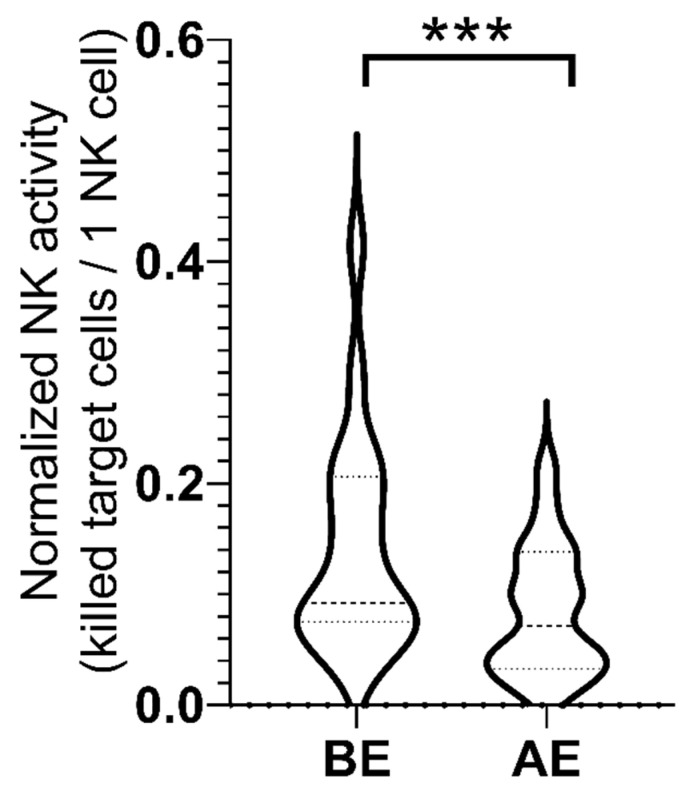
NK cell function. NK cells were co-incubated with K562 cells at 20:1 effector/target ratio for 2 h at 37 °C. The percentage of killed K562 cells were analyzed. The NK activity values were normalized to the absolute number of NK cells (number of killed K562 target cells/number of NK cells in the sample). *** *p*  <  0.001 (Wilcoxon test).

The normalized NK cell activity decreased due to our exercise protocol in the group (BE: 0.14 ± 0.02, AE: 0.08 ± 0.01) (Figure 2). Our results indicate that the 43% decrease in the values after the exercise protocol was significant (*p* < 0.05, ES = 0.941).

## 3. Discussion

The findings of our study show an increase in the frequency of CD3^−^/CD56^+^ NK cells among lymphocytes due to our exercise protocol. This result is in line with earlier findings [26,27]. The same exercise-induced increase in frequency could be observed both in the case of CD3^−^/CD56^bright+^ and CD3^−^/CD56^dim+^ NK cells. It is noteworthy that the finding of increased CD3^−^/CD56^dim+^ NK cells due to our exercise protocol also seems to be in line with earlier findings [26]. No significant change of CD3^−^/CD56^bright+^ NK cell frequency among lymphocytes was detected.

The NK cells showed significantly decreased activity after our exercise protocol. Post-exercise depression in NK cell activity has been demonstrated, especially after intense exercise [28].

The percentage of CD3^+^ T cells among lymphocytes showed a decrease due to our exercise protocol. That is again in line with the earlier findings of Pizza et al. [27].

It is important to highlight that the above opposing trends were all independent of the DOMS effect. On the other hand, the percentage of NKT-like cells among lymphocytes exhibited a different pattern, namely, DOMS induced an imbalanced control of these cells as opposed to participants without the DOMS effect. Correspondingly, it seemed that exercise in the absence of muscle spindle microdamage did not impact the percentage of NKT-like cells among lymphocytes and they maintained a relatively steady ratio. DOMS could alter this homeostasis by significantly increasing the percentage of NKT-like cells; however, in some cases, it decreased their ratio among lymphocytes. It must be noted that Pizza et al. showed earlier that downhill running significantly increased the total number of NKT-like cells post-exercise in contrast to level running [27].

NKT-like cells share both NK and T cell features and are involved in both innate and acquired immune responses. They comprise 5–15% of the overall peripheral T cell population [29,30]. Acute exercise increases the expression of cellular protective proteins in lymphocytes such as heat shock protein 70 (Hsp70) in NK cells, T cells, and NKT-like cells [31]. Furthermore, the extracellular-to-intracellular ratio of Hsp70 increases due to acute exercise [31]. It is interesting to note that NKT-like cells are dissimilar among T cells in the expression level of these cellular protective proteins such as Hsp70, and they seem to be more sensitive to alterations [32]. Indeed, our findings show that the percentage of NKT-like cells among lymphocytes is relatively resistant to ratio alterations during exercise, while other NK cells increase, but DOMS leads to imbalanced control of this ratio measured immediately post-exercise.

Hsp70 is a very conserved stress protein with a role in the protein quality control system and the regulation of the NF-κB pathway [33]. It is noteworthy that Hsp70, released due to DOMS-inducing exercise, activates the TLR4/Interleukin-6/TNF-α pathway in the spinal cord [34]. It is important to highlight that Hsp70 has opposing effects, namely, anti-inflammatory intracellularly and rather pro-inflammatory extracellularly [35]. Accordingly, intracellular Hsp70 is anti-inflammatory by blocking NF-κB activation, while extracellular Hsp70 can activate the NF-κB pathway extracellularly through binding to TLR4 [35,36]. The activated NF-κB pathway suppresses autophagy, hence guiding the way to inflammation and immune activation [35,37,38]. It is noteworthy that DOMS indeed induces spinal cord NF-κB activation [16]. In fact, the intrathecal treatment with NF-κB and glial inhibitors could rectify all the immune and cellular alterations and resultant neuroinflammation induced by DOMS [16]. The current authors even propose that DOMS-inducing exercise also increases the surface Hsp70 on the extracellular surface of plasma membranes in a similar fashion as experienced in tumor cells without a pro-tumor manner [39]. It’s important to note that surface Hsp70 also serves as a recognition pattern for the immune system [40]. Correspondingly, the authors suggest that the acute exercise-derived stress response (ASR) in DOMS induces such a surface Hsp70 recognition pattern that attracts NKT-like cells distinctly, similarly to what we could see in tumors in a MHC-unrestricted fashion [41]. It is noteworthy that the surface localization of Hsp70 is membrane cholesterol-dependent [42]; however, the suggested transient Piezo2 microinjury is associated with membrane cholesterol depletion around the ion channel [43,44,45]. Therefore, the lack of membrane cholesterol rafts could be one explanation for the imbalanced regulation of NKT-like cells. Notably, one consequence of surface Hsp70 expression in tumors is derailed endocytosis [46]. Moreover, impaired vesicular glutamate transport at the primary afferent terminal in the muscle spindle is theorized to be part of the DOMS pathomechanism [18,21]. Accordingly, the current authors propose that vesicular glutamate transport becomes impaired due to an analogous derailed endocytosis effect in DOMS at the Ia proprioceptive terminal.

Therefore, the activated Hsp70/TLR4/Interleukin-6/TNF-α pathway could play a crucial role in the imbalanced control of CD3^+^/CD56^+^ NKT-like cells in DOMS. Furthermore, the activation of the Hsp70/TLR4/Interleukin-6/TNF-α pathway could be distinct to the somatosensory terminal microinjury that contributes to proprioception, as in DOMS.

It is noticeable that the critical injury of DOMS is hypothesized to be a terminal arbor degeneration (TAD) such as lesions at the primary proprioception terminal in the muscle spindle [9], as could be experienced in paclitaxel chemotherapy [17]. According to the TAD theory, “if the energy deficiency is severe enough then degeneration happens, and the threshold for degeneration will be lowest in the neuronal compartment that has the highest energy requirement” [17]. It was suggested that the Type Ia proprioceptive terminal in the muscle spindle is an analog compartment with the highest energy requirement in the DOMS mechanism [9]. This type of microdamage could occur in an acute and chronic fashion without Wallerian degeneration [17,47]. Indeed, it was shown that this type of complex Type Ia proprioceptive impairment could evolve after platinum analogue chemotherapy [47,48]. Furthermore, it was also demonstrated that a chronic central synaptic disconnection of proprioceptors from motoneurons could evolve after nerve injury due to the loss of vesicular glutamate transporter (VGLUT) 1/Ia synapses on motoneurons [49,50]. It is noteworthy that paclitaxel, the aforementioned TAD causing chemotheraputic agent, potentiates Piezo2 currents [51], not to mention that Piezo2 is found to be the primary mechanotransduction channels for propriocpetion [52].

Information about the targeted delivery of cytotoxic factors through the immune synapse along Wallerian degeneration is emerging [53], but not in reference to TAD-like lesions. Furthermore, it is theorized that this type of non-contact mechano-energetic microinjury could evolve on Piezo2 ion channels of somatosensory endings contributing to proprioception [18]. This autologous microlesion is devoted to an ASR-derived mechano-energetic impairment of Piezo2 channels and glutamate vesicular release [18]. One consequence of this TAD-like lesion in DOMS is suggested to be a loss of vesicular glutamate transporter (VGLUT) 1/Ia monosynaptic connection on motoneurons and resultant N-methyl-D-aspartate (NMDA) receptor activation on the spinal dorsal horn [54], as was demonstrated after nerve injury [49,50]. Hence, the current authors suggest that the activation of the Hsp70/TLR4/Interleukin-6/TNF-α pathway is associated with transient Piezo2 channelopathy on somatosensory terminals contributing to proprioception, similarly to the way it is experienced in DOMS. Indeed, it seems that a distinct cross-modulatory mechanism could exist between Piezo2 microinjury and interleukin-6 [55,56].

It must be noted that the autoreactive activation of NKT-like cells is not entirely understood [57]. However, the ASR-derived impairment of glutamate vesicular release-based autoexcitation of muscle spindles could be a possible clue to how NKT-like cells, also called innate-like cells, go through autoreactive activation. Furthermore, the proposed imbalanced subthreshold calcium currents due to “leaky” microinjured Piezo2 channels is in line with the findings that impaired calcium signaling is part of the autoreactive activation of NKT-like cells [45,57].

DOMS is considered a bi-phasic injury mechanism [9,58,59]. The acute non-contact compression axonopathy theory of DOMS postulated that the first phase of DOMS is the aforementioned microinjury of the primary afferent terminal in the muscle spindle. This hypothesis also comprised an intimate crosstalk between the involved somatosensory fibers. The link in this cross-communication is suggested to be Piezo2 ion channel-based [12,45]. Polimodal Type III/IV nociceptors expressing Piezo2 could be activated by mechanical stimuli, heat, and chemical irritants [45]. Accordingly, in the second phase of DOMS, Type III or Aδ sensory afferent neurons could go through metabolic/enzymatic activation [20]. Metabolites produced by exercise could activate Type III/IV sensory neurons, leading to fatigue and muscle pain [60]. Interestingly, these metabolites at low levels contribute to the perception of fatigue, while at high levels, to the perception of pain [60]. Indeed, enzymatic destruction of the extracellular matrix (ECM) impairs Piezo2 mechanogating [61]. This noxious chemical stimuli in DOMS could take place in the deep fascia or in skeletal muscles [12,62]. Furthermore, recent findings show that nerve growth factor (NGF)-tropomyosin receptor kinase A (TrkA)-Piezo2 signaling axis also has a role on Aδ sensory afferent neurons in noxious mechanical stimulation [12,45,63]. Piezo2 is even a contributor to the sensitization of these neurons to mechanical stimulation [63]. However, the current authors propose that this secondary sensitization only takes place in the case of transient Piezo2 channelopathy present in the first injury phase of DOMS.

It is noteworthy that data are emerging on how ECM proteins regulate NK cell function in peripheral tissues [64]. The current authors suggest that the aforementioned DOMS-induced enzymatic destruction of ECM also has a role in the observed imbalanced control of NKT-like cells. It is possible that the proposed transient Piezo2 channelopathy-induced interleukin-6 activates NKT-like cells in a similar TrkA-dependent activation mechanism in the ECM, similar to the way IL-2 activates NK cells [65]. In addition, TLR4 might have a cross-signaling role as well in the ECM beyond the NGF-TrkA signaling mechanism. Indeed, Piezo1 enhances LTR4-mediated innate immune response [66]. Furthermore, NMDA receptor activation in the ECM might play a role, as well [67].

Finally, a recently published paper theorized that an analogous metabolic machinery of astrocyte-neuron lactate shuttle could be present at the Type Ia proprioceptive terminals in cooperation with intrafusal satellite cells, like in the central nervous system [68]. Moreover, DOMS is assumed to be associated with impairment of this lactate shuttle machinery [68]. A high-lactate environment is not advantageous for NKT-like cells’ survival and proliferation, but glutamine is essential [69]. Glutamine is released into the extracellular space due to the lactate shuttle machinery and is taken up by neurons [70,71,72]. However, the DOMS-associated malfunction of this lactate shuttle machinery, most likely due to the dysfunctional mitochondria-induced impairment of glutamate vesicular release at the Type Ia proprioceptive terminal [18,54], could lead to a higher glutamine presence in the extracellular space, and that could be an additional metabolic reason for NKT-like cell attraction, especially if the distinct metabolic programing of these cells is considered [69].

### Limitations

The low number of subjects who did not report DOMS is acknowledged; however, this number could not be projected prior to the execution of the exercise protocol. Furthermore, the potential source of bias should be considered due to the homogenous population sample, and the sample represented a minority of the population. Correspondingly, the authors found it important to highlight observations in reference to the differences between the DOMS-reporting group and the one without DOMS, despite of the low sample size of the latter group. It is important to note that this study could lay the foundation for a larger sample size study in the future.

## 4. Materials and Methods

### 4.1. Study Design

In this prospective observational study, we performed sports cardiology screenings on members of the youth Hungarian National Swim Team in March 2022. The study was accepted by the Hungarian Central Ethics Committee (ETT TUKEB IV/10282-1/2020/EKU) and the Semmelweis University Regional and Institutional Committee of Science and Research Ethics (SE RKEB 282/2021). All participants signed informed consent on written forms.

### 4.2. Participants

We included 19 youth swimmers, 10 females and 9 males, into the study with a mean age of 16.5 years. All participants were members of the National Swim Team of Hungary with qualification for the European Championships. All athletes were in the same so-called preparation phase of the training during our examination (Table 2). Athletes were instructed to do their morning routine before the examination, including having breakfast and staying hydrated.

### 4.3. Procedures

#### 4.3.1. Sports Cardiology Screening with Wall-Sit Test

We performed a detailed sports cardiology screening on all athletes including a sports-specific questionnaire, laboratory test, resting electrocardiogram (ECG), echocardiography, body composition analysis, and cardiopulmonary exercise test (CPET) at prescheduled dates. The examinations were delivered by the same medical doctors and operators. After the CPET exam, all athletes were instructed to hold a 5 kg weight in the wall-sit position in order to provide an isometric, static load for muscles to reach maximal fatigue in the lower extremities. Athletes stayed in this wall-sit position until exhaustion.

Personal history and symptoms were assessed on a sports-specific questionnaire. We specifically asked for DOMS experience before the exercise and the level of pain was assessed 24 h and 48 h post-exercise. We used the visual analog scale (VAS 1-10) as a tool for self-reported assessment to quantify DOMS. Standard resting 12-lead ECGs were recorded by CardioSoft PC (GE Healthcare, Finland). Laboratory tests were performed before and after the isometric weight holding task. We examined from the venous blood samples the qualitative and quantitative blood counts, high-sensitive troponin T, creatine kinase, creatine kinase-MB, and NK cell activity. Echocardiography exams were performed on a GE Vingmed Ultrasound E95 Ultrasound system (4Vc-D probe, Horten, Norway). Cardiorespiratory fitness was determined by a treadmill ergometer (GE T-2100, Healthcare, Finland). The exercise protocol was the same as we described in our earlier study [73]. It contained a 1-min preparation phase in standing position, a 2-min walking warm-up phase (6 km/h), a running phase (8 km/h) with progressive increment of inclination rate of 1.5% every 2 min until exhaustion, and a recovery period with 1-min active walking recovery and 4-min passive recovery. Athletes were instructed not to hold the handrail. Gas parameters were calculated by a breath-by-breath automated cardiopulmonary exercise system (Respiratory Ergostik, Geratherm, Bad Kissingen, Germany). Athletes were encouraged to reach maximal exertion, which was confirmed by respiratory exchange ratio, and by reaching the predicted maximal heart rate and VO_2_ value [73].

#### 4.3.2. Natural Killer Cell Activity

NK cell activity was evaluated by flow cytometry [74,75]. K562 human chronic myelogenous leukemia cells (ATCC; Manassas, VA, USA) were used as targets in the assay. K562 cells were cultured in complete cell culture medium consisting of RPMI-1640 Medium (Sigma, St. Louis, MI, USA) supplemented with 10% fetal bovine serum, 100 U/mL penicillin, and 100 mg/mL streptomycin (Gibco BRL, Grand Island, NY, USA). Target and effector cells (PBMC) were incubated together at 1:20 ratio for 2 h at 37 °C. Annexin V staining and propidium iodide uptake were used to detect cytolysis of K562 cells.

#### 4.3.3. PBMC Seperation

Histopaque^®^-1077 (Sigma, St. Louis, MI, USA), a density gradient cell separation medium, was used for the separation of peripheral blood mononuclear cells (PBMC) under sterile conditions, as recommended by the supplier.

#### 4.3.4. Immunophenotyping of Circulating NK Cells

One hundred μL of anticoagulated whole blood was incubated with a pre-titrated optimal concentration of R-Phycoerythrin (Pe)-conjugated anti-human CD56 and Alexa Fluor 488-conjugated anti-human CD3 monoclonal antibodies (15 min, room temperature) for the identification of T, NK, and NKT lymphocytes. After the incubation, red blood cells were lysed by FACSLysing solution (BD Biosciences, San Jose, CA, USA) according to the instructions of the manufacturer. After erythrolysis, cells were washed twice and were fixed in 300 μL of 2% paraformaldehyde solution (Sigma). Stained cells were stored at 4 °C in the dark before analysis. Both fluorochrome-conjugated monoclonal antibodies were purchased from Sony Biotechnology Inc. (San Jose, CA, USA). Tests were carried out by measuring 5 × 10^4^ cells/sample on the day of the staining.

#### 4.3.5. Flow Cytometry Measurements

Measurements were carried out using a FACSCalibur flow cytometer (Becton Dickinson San Jose, CA, USA) on the day of sample preparation. CellQuest-Pro software (Becton Dickinson San Jose, CA, USA) was used for acquisition and analysis.

### 4.4. Statistical Methods

The basic data were characterized by means and standard deviations. To determine normality, because of the limited sample size and for the purpose of selecting the adequate statistical procedure, Shapiro Wilk’s W test was applied. As our research model was based on a before–after comparison, differences between the datasets were compared with a dependent t-test when the data was normally distributed, and if this was not the case, a Wilcoxon test was applied. To further support the results of this study, we calculated and included effect size (ES-Cohen’s d) values. A correlation calculation was performed applying a Chi-square 2 × 2 contingency table. Data analysis was performed on Statistica 12 program (Statsoft Statistica, TIBCO Software Inc., Palo Alto, CA, USA); the significance level was determined to be *p* < 0.05.

## Figures and Tables

**Table 1:** Average ± SE values for the indicated percentage variables.

Analyte	Before Exercise (BE)	After Exercise (AE)	Significance
CD3^−^/CD56^+^ NK cells (% inside lymphocyte gate ± SE)	12.60 ± 1.01	20.96 ± 2.04	*p* = 0.0001
CD3^+^/CD56^+^ NKT-like cells (% inside lymphocyte gate ± SE)	4.13 ± 0.59	6.14 ± 0.98	*p* = 0.0004
CD3^−^/CD56^dim+^ NK cell subset (% inside lymphocyte gate ± SE)	11.69 ± 1.04	19.79 ± 2.05	*p* = 0.0002
CD3^−^/CD56^bright+^ NK cell subset (% inside lymphocyte gate ± SE)	0.91 ± 0.09	1.16 ± 0.10	n.s. (*p* = 0.1)
Normalized NK cell activity (killed target cells/1 NK cell ± SE)	0.14 ± 0.02	0.08 ± 0.01	*p* = 0.0009
CD3^+^ T cells (% inside lymphocyte gate ± SE)	65.54 ± 1.53	58.03 ± 2.02	*p* < 0.0001

**Table 2:** Baseline characteristics of the 19 athletes involved in the study.

Age (years)	16.5 ± 2.8
Female (*n*,%)	10 (53%)
Training (hours/week)	21 ± 2.5
Training History (years)	11.7 ± 3.2
Last Training before Exam (h)	22.7 ± 14.1
Last Training Length (min)	105 ± 39.5
Wall-Sit Time (s)	127.1 ± 46.2
Muscle Fever Pre-Exercise (VAS 1-10)	1.42 ± 2.0
Muscle Fever Post-Exercise 1th day (VAS 1-10)	3.2 ± 2.6
Muscle Fever Post-Exercise 2nd day (VAS 1-10)	2.9 ±2.5
CK before Exercise (U/L)	202 ± 125
CK after Exercise (U/L)	219 ± 123
CKMB before Exercise (U/L)	18 ± 3.9
CKMB after Exercise (U/L)	19 ± 3.3
Troponin T before Exercise (ng/mL)	9.4 ± 8.0
Troponin T after Exercise (ng/mL)	9.5 ± 7.4
Load Time (min)	13.4 ± 1.1
Peak Lactate (mmol/L)	10.4 ± 2.6
Restitution Lactate (mmol/L)	9.6 ± 2.7
VO_2_ Max Male (mL/kg/min)	58.2 ± 1.8
VO_2_ Max Female (mL/kg/min)	53.4 ± 3.1
VO_2_ Max (mL/kg/min)	55.1 ± 3.9

Legend–Average ± SD; CK: creatine kinase, CKMB: creatine kinase MB; VO_2_ max: maximal aerobic capacity.

## Data Availability

The data presented in this study are available on request from the corresponding author.
